# Burden of herpes zoster and post-herpetic neuralgia in Sweden

**DOI:** 10.1186/s12879-015-0951-7

**Published:** 2015-05-22

**Authors:** Jonas Nilsson, Tobias Cassel, Lars Lindquist

**Affiliations:** Mapi Group, Stockholm, Klarabergsviadukten 90D, SE-111 64, Stockholm, Sweden; Sanofi Pasteur MSD, Solna, Sweden; Karolinska Institutet, Department of Medicine, Huddinge, Sweden

## Abstract

**Background:**

The societal economic burden of herpes zoster in Sweden is not well described today. This study is a top-down analysis of Swedish registers with the objective to describe the burden of herpes zoster and post-herpetic neuralgia in Sweden during 2011.

**Methods:**

Data for inpatient care; outpatient primary and specialized cares; the prescriptions of drugs, sick leave and the number or diagnostic tests were collected from Swedish national databases. The incidence of the disease was estimated based on the number of prescriptions of antiviral drugs.

**Results:**

The incidence of herpes zoster was estimated to 315 and 577 cases per 100,000 people for patients at all ages and > = 50 years, respectively. Almost 30,000 patients at all ages were diagnosed with herpes zoster and the societal cost to treat these patients, including the cost to treat those patients who later developed post-herpetic neuralgia, added up to nearly 227 MSEK (31.6 M€) which corresponds to 7,600 SEK (€870) per patient. The main contributors to the total cost for the treatment of HZ patients were primary care (43 %); sick leave (28 %); hospitalization (10 %) and specialist care (7 %). Medication was a relatively small contributor with 8.5 MSEK (4 %; 1.0 M€) to the overall costs for patients at all ages. The corresponding total cost including only patients 50 years and older was 168 MSEK (19.2 M€) or 8,200 SEK (€939) per patient.

**Conclusions:**

The current study demonstrates that the burden of herpes zoster is significant in Sweden. The society, the health care payers and the patients potentially have a lot to gain by introducing a vaccination program to patients 50 years and older and as a consequence reduce the economic and clinical burden of herpes zoster and post-herpetic neuralgia.

## Background

Herpes zoster (HZ), also known as shingles, results from reactivation of latent varicella-zoster virus (VZV) infection residing in the spinal and cranial sensory ganglia. The initial infection of VZV usually occurs during childhood and manifests clinically as chickenpox. HZ as a subsequent reactivation, more seldom as a result of reinfection, of VZV presents as a clinical syndrome characterized by a unilateral radicular pain and avesicular, distributed cutaneous rash [1]. The annual HZ rate in the general population has been estimated to be 3–6 cases per 1,000 person-years in the United States (US), the United Kingdom (UK) and Europe [2–6], and the estimated average lifetime risk of HZ was approximately 30 % reported from UK and Canadian studies [7]. The incidence and severity of HZ increase markedly with advancing age, about half of all cases occur amongst individuals 60 years old or older. In Sweden, a recent burden of illness study on HZ conducted by Studahl et al. [8] found similar results by analyzing data from Swedish national registries during 2006–2010. It reported that 2.7 per 1,000 persons were prescribed antiviral treatment for HZ every year and the prescription rate increased with age [8]. The overall hospitalization rate for HZ patients over 80 years was approximately three-fold higher than for HZ patients aged 70–79 years [8].

The most frequent complication of HZ is post-herpetic neuralgia (PHN) usually defined as a neuropathic pain syndrome that persists or develops more than three months after the acute HZ infection and dermatomal rash have healed [1, 9]. The frequency and severity of PHN also increase with advancing age. Among individuals who have had acute HZ, it is estimated that about 20 % of those aged 60 years or older and more than 30 % of those over 80 years develop PHN [9]. Approximately 90 % of these complain that pain is a predominant symptom, often refractory to treatment, and about 2 % may suffer from pain for many years [9, 10]. The burden of HZ and PHN placed on health care systems is therefore considerable.

The burden of HZ in Sweden from a clinical perspective has been discussed in a previous study by Studahl et al. [8] for the years 2006–2010. The objective of the current study is to assess the burden and societal costs for HZ and PHN in Sweden during 2011.

## Methods

Data that has been analyzed in the current study were taken from Swedish national databases and were not based on patient level data; hence, an ethical approval was not needed and no additional permission was needed. Costs were presented as 2013 Swedish *kronor* (SEK), key costs ware also given in Euros within brackets. Costs were inflated to 2013 using the Consumer Price Index (CPI) taken from Statistics Sweden where necessary; Conversion rate SEK to Euros average during 2013: 8.6494], and the resource utilization was summarized for the year 2011. All costs and values obtained from the Swedish national databases were provided as unweighted means based on aggregated data across 2011. Information regarding the dispersion around the unweighted mean was not available and, therefore, the uncertainty will be tested in sensitivity analysis (see below).

### Target population

The main analyses presented in the current study focus on the costs for patients at all ages diagnosed with HZ and PHN. The age dependent incidence of VZV infections manifested as chickenpox versus HZ has been described by Yih et al. [11], Jumaan et al. [12] and Péréz-Farinos et al. [13]. Based on these studies, and the fact that only patients 50 years and older are eligible for vaccination in Sweden, it was also of interest to present the cost and incidence specifically for these patients in addition to the costs and incidence for all patients.

### HZ and PHN Incidence

The incidence of HZ was estimated from the number of prescriptions of antiviral drugs with specific package sizes as described in Table 1. Prescriptions were obtained from the Swedish National Pharmacy Register (SNPR) covering close to 100 % of the prescribed medications. However, the number of antiviral prescriptions was corrected slightly since not only HZ patients are prescribed antiviral medications (see section Drug treatment below). The incidence reported by Studahl et al. [8] was from 2006–2010 but was updated with data from 2011 in this study. The share of HZ patients who developed PHN was taken from Gauthier et al. [14]. Here the share of patients, who were diagnosed with HZ and developed PHN within three months in the UK, was used as the incidence of PHN also in Sweden. The incidence was reported as the number of patients who were diagnosed with HZ or PHN per 100,000 Swedish people during 2011.

**Table 1:** Antiviral medications and specific packages^§^ used for the treatment of HZ

Drugs	ATC^*^	DDD^**^	Days	Package size
Aciclovir	J05AB01	800mg × 5	7	35
Famciclovir	J05AB09	500mg × 3	7	21
Valaciclovir	J05AB11	500mg × 2 × 3	7	42

*ATC = Anatomic Therapeutic Chemical; **DDD = Defined Daily Dose; ^§^The same package sizes were also used by Studahl et al. ([8] and personal communication)

### Mortality

Deaths related to HZ (ICD-10: B02) were collected from the Swedish Causes of Death Register covering all the people who died and were registered as living in Sweden until 2011.

### Inpatient care

The Swedish Association of Local Authorities and Regions [15] are conducting a country-wide project to support the calculation of patient-related costs of care. As a result, the cost per patient for the treatment of various diagnoses is publically available via the KPP (KPP stands for cost per patient) database [15]. Although the KPP database does not cover the entire country yet, the costs for the treatment of HZ (DRG = 421) and PHN (DRG = 020) were taken from this database.

The number of inpatient treatments of HZ as primary cause (ICD-10: B02) and PHN as primary cause (ICD-10: G53.0), as well as the average duration of hospitalization, were taken from the Swedish National Patient Registry (held by the Swedish Board of Health and Welfare).

### Outpatient specialist care

Statistics covering specialist care performed by physicians who are not general practitioners (GPs), are available from the Swedish National Patient Registry. The number of outpatient visits to specialist care with HZ or PHN as a primary or secondary diagnosis during 2011 was included in the analyses. A visit to a specialist was assumed to cost as a visit to an average specialist (average of the cost from visiting an ophthalmologist, neurologist, dermatologist, pain specialist or an infection specialist). Unit costs for the visits to the different types of care were derived as the average cost from the different regions and county councils in Sweden [16–20] as presented in Table 2.

**Table 2:** Cost per outpatient visit to primary and specialist cares

Care	Average cost per visit (SEK) (SD)	HZ:Number of visits; all ages (> = 50 years)	PHN: Number of visits; all ages (> = 50 years)	Source
Outpatient care (primary)				
*General practitioner (GP)*	1 544 (214)	47 047 (35 479)	882 (799)	[17, 18, 20]
*Physician on call*	2 316 (321)	10 534 (6 904)	12 (0)	50 % higher than GP visit [16]
Home care*	422 (NA)	1 848 (1 765)	30 (30)	[19]
Outpatient care (specialist)				
Specialist visit**	2 399 (732)	6 517 (5 292)	617 (570)	Average of cost to specialists**

*Primary care organized home care; **Average costs (ophthalmologist, neurologist, dermatologist, pain specialist, infectious specialist) calculated from pricelist in Norrland county council, Västra Sjukvårdsregionen, Sahlgrenska hospital, Stockholm and Gotland county council, Linköping county council and Södra Sjukvårdsregionen

### Outpatient primary care

Primary care data for HZ and PHN patients during 2011 were extracted from Västra Götaland county council (VGR) and scaled to the entire Swedish population based on the share of the population the VGR county council is covering, which currently is 16.8 % (1.59 M people) of the Swedish population. The VGR register all care (primary and specialist) consumed by its population in a local database. Primary care predominantly involved visits to general practitioners, doctors on call or home care visits. All the unit costs are listed in Table 2. The cost for a physician on call was estimated to be 50 % higher than a regular visit to a GP as suggested in the pricelist from South-East Sweden [16].

### Diagnostic tests

Herpes Zoster is predominately diagnosed based on the clinical situation; however, a couple of diagnostic tests are being used for patients with HZ or suspected HZ. The number of diagnostic tests performed with HZ patients was available in the Stockholm county council (Faktureringsunderlag Medicinsk Service (FUMS)). The tests used for HZ patients included IgG, a serologic laboratory test used to identify previous exposure to VZV and immunity, and second, measuring the presence of DNA in blisters or used as a laboratory diagnostic test of VZV-infected patients. The total number of tests performed in Sweden during 2011 was derived based on the share of the total population covered by the Stockholm county council, which corresponds to 22.1 % (2.09 M people). The cost per diagnostic test (940 SEK (€109)) including IgG and DNA was taken from the list price at a commercial vendor [21].

### Drug treatments

Prescriptions of drugs to patients at all ages and > =50 years, during 2011 were extracted from the SNPR. In general, HZ patients are prescribed antiviral drugs only in the package sizes listed in Table 1, with enough pills to cover seven days of treatment. Therefore, the corresponding number of patients was used as a measure of the incidence of HZ during 2011 [8]. Antiviral drugs can also be prescribed to patients with other infections than VZV, e.g., primary infections with VZV, as outlined by Studahl et al. [8]. To correct for this, prescriptions with the words: simplex, genital, mouth, labial, varicella, prophylaxis or chickenpox in the patient information field were excluded, amounting to 12 % of the antiviral prescriptions [8].

Among those patients who were prescribed antiviral drugs, other drugs related to the treatment of HZ and PHN were also collected, including non-selective monoamine re-uptake inhibitors (e.g., amitryptiline), antileptics (e.g., gabapentin and pregabalin), opiods (e.g., tramadol) and anti-depressants (e.g., venlafaxine). As the number of prescriptions of other drugs than antiviral drugs was based on the same patients who were prescribed antiviral drugs, the number of prescriptions of other drugs was adjusted downwards by 12 % to correct for the patients with other infections than VZV (see above).

The cost per package was assumed to be equal to the cheapest competitor in case no price was available in Farmaceutiska Specialiteter i Sverige (FASS) [22].

### Sick leave

Data regarding sick leave for patients who were off sick more than 14 days due to HZ/PHN during 2011 were obtained from the Swedish Social Insurance Agency (SSIA). Sick leaves shorter than or equal to 14 days are not registered by the SSIA since employers are responsible for the payment of sickness benefits during the first two weeks of sick leave. Therefore, the number of days of sick leave below this threshold was estimated based on data in a previous study. Drolet et al. [23] found that the majority of employed HZ patients (64 %) reported an average absence from work of 27 h during the first 30 days of sickness. White et al. [24] found that HZ patients (18–65 years) in the US were on sick leave on average 26.5 h the first year after diagnosis; no specification on how these hours were distributed during the index year was provided in the study. In a telephone survey performed in the US by Singhal et al. [25] it was found that patients between 50–64 years were on average away from work 31.6 h per HZ episode. In those cases where the duration of the HZ episode was less than a month, patients lost on average 12.1 h of work. In addition to the absenteeism (time away from work), presenteeism-related work loss (for those who go to work despite their illness) could not be estimated but could add to the sick-leave related work loss as shown by Drolet et al. [23] and Singhal et al. [25].

In the current study, the work loss reported by Drolet et al. [23] (27 h the first 30 days from index date) was used as an estimation of the work loss for Swedish HZ patients during the first 13 days of illness. Thus, patients were off sick on the average 3.38 days (assume eight hours of work per day; 27/8 = 3.38) if the sickness period was less than 14 days.

The number of employed HZ patients was calculated based on the employment rate (per 10-year age groups from 15 to 74 years old) , including both males and females, in Sweden during 2011 obtained from Statistics Sweden [26].

To calculate the cost for lost production for patients on sick leave the average monthly salary was obtained from Statistics Sweden [26]. The daily wage was derived assuming 40 h of work per week and an employer’s contribution of 31.42 % [27] yielding 1,742 SEK (€201) per day.

### Sensitivity analysis

Parameters that were estimated rather than obtained from any registers, i.e., the number of sick leave days shorter than 14 days, the GP consultation cost, daily hospitalization cost and the cost of drugs, were included in one-way sensitivity analyses experiments. The base case values were increased with 25 % for all parameters except for the number of short term sick leave days that was increased with 50 %. The impact on the total cost of HZ, including patients at all ages, was estimated for each experiment.

## Results

### Incidence

**Fig. 1:**
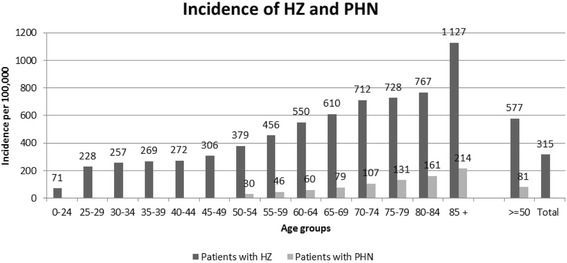
The incidence of herpes zoster (HZ) and post-herpetic neuralgia (PHN) during 2011 (incidence per 100,000 persons)

The incidence of HZ during 2011 is presented per age group in Fig. 1. In total, 29,900 (all ages) and 2,860 patients (> = 50 years) were diagnosed with HZ and PHN in Sweden during 2011, respectively. The corresponding number for patients 50 years and older with a diagnosis of HZ was 20,446. The incidence of HZ is clearly higher for the group of patients older than 50 years compared to the whole population with 577 and 315 cases per 100,000 persons, respectively (Fig. 1). The incidence of PHN was only estimated for patients older than 50 years to 81 cases per 100,000 persons.

**Fig. 2:**
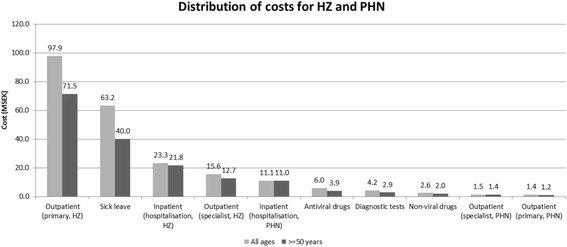
Break down of costs for the burden of HZ and PHN in Sweden 2011 for all ages (grey bars) and > =50 years (black bars)

### Mortality

Only eleven people died from HZ in Sweden during 2011 (0.12 per 100,000 people) and it was assumed that death did not contribute with any significant cost to the overall burden of HZ.

### Health care utilization

In Table 3, the number of visits to the different types of care utilized for the treatment of HZ and PHN are summarized, including inpatient and outpatient (primary and specialist) care. Clearly, the dominating medical resource use for HZ was outpatient visits to a physician with almost 60,000 visits followed by outpatient visits to a specialist with 6,500 visits in 2011. The number of visits is real world data and, therefore, each visit will incur a cost regardless if a patient contributed with more than one visit.

**Table 3:** Number of health care visits for HZ and PHN patients during 2011 including: inpatient care (hospitalization), outpatient care (primary) and outpatient care (specialist)

Type of care	Diagnosis	Number of visits; all ages (> = 50 years)	Total cost; all ages (> = 50 years) (SEK)
Inpatient care (hospitalization)*	HZ	725 (640)	23 342 200 (21 784 000)
	PHN	222 (217)	11 107 400 (11 014 000)
Outpatient care (primary) **	HZ	59 457 (44 147)	97 876 300 (71 524 300)
	PHN	924 (829)	1 402 800 (1 246 300)
Outpatient care (specialist)*	HZ	6 517 (5292)	15 634 300 (12 695 500)
	PHN	617 (570)	1 480 200 (1 367 400)
Total:			150 843 200 (119 631 400)

*Swedish National Patient Registry; **Numbers from Västra Götaland County Council scaled to Sweden based on share of the population

From the KPP database the costs per HZ and PHN hospitalizations were taken and equaled 41,000 SEK (€4,800) and 32,000 SEK (€3,700), corresponding to 7.87 and 5.44 days of hospitalization, respectively. The daily hospitalization costs were calculated for HZ and PHN to 5,261 SEK (€608) and 5,840 SEK (€675), respectively. The average hospitalization time for patients older than 50 years was calculated to 6.47 days and 8.69 days for HZ and PHN, respectively, based on data from the Swedish National Patient Registry. Note, that the number of hospitalization days, used to estimate the total hospitalization costs, were taken from the Swedish National Patient Registry, rather than the KPP-database, as the former was based on all registered hospitalisations. Only a small part 2.7 % and 5.5 % of the HZ and PHN patients 50 years and older, respectively, were hospitalized while incurred 13 % of the total costs.

The treatment of HZ patients incurs clearly more resource use than the treatment of PHN patients with estimated costs for hospitalization of 23 and 11 MSEK (2.7 and 1.3 M€), primary care of 98 and 1 MSEK (11 and 0.1 M€) and specialist care of 16 and 1 MSEK (1.8 and 0.1 M€), respectively (Fig. 2). The share of hospitalization costs for PHN patients was higher relative to the cost for HZ patients as compared to the corresponding costs for the outpatient cares.

The overall health care costs for HZ and PHN patients add up to over 151 MSEK (17.6 M€) where the cost for primary care is dominating. For patients 50 years and older the corresponding cost is 120 MSEK (14 M€) which is 79 % of all the health care costs.

### Diagnostic tests

In Sweden, the total cost for diagnostic tests performed to establish the HZ diagnosis was estimated to around 4 MSEK (0.5 M€; IgG and DNA with 3 MSEK and 1 MSEK, respectively) during 2011 based on 3,686 and 812 numbers of tests for IgG and DNA, respectively. Including only patients > = 50 years the total cost was almost 3 MSEK (0.3 M€).

### Drug treatments

The total costs of drug treatment were derived from the number of prescriptions of antiviral drugs and non-viral drugs to HZ/PHN patients and the cost per prescription [22] (Table 4). In total, the antiviral drugs and non-viral drugs contributed with around 8.5 MSEK (0.99 M€) to the overall burden of HZ in 2011 in all ages. Out of the 6 MSEK (0.7 M€) that was spent on anti-viral drugs, 72 %, 24 % and 5 % were spent on valaciclovir, aciclovir and famciclovir, respectively. Non-viral drugs included amitryptiline (mono-amine reuptake inhibitors), gabapentin and pregabalin, tramadol (opioids) and venlafaxine (antidepressant). Patients 50 years and older accounted for 69 % (5.9 MSEK; 0.7 M€) of the total drug cost.

**Table 4:** Costs for drugs prescribed to HZ patients at all ages during 2011. In parenthesis prescriptions for patients > =50 years

Drug	Number of prescriptions, all ages (> = 50 years)^*^	Total cost all ages (> = 50 years) (SEK)
Antiviral	32 640 (22 195)	5 980 800 (3 925 200)
Non-viral	22 734 (18 022)	2 577 500 (1 962 600)
Total:		8 558 300 (5 887 800)

*The number of prescriptions was adjusted downwards with 12 % to correct for prescriptions to other diagnosis than HZ

### Sick leave

During 2011, 370 patients with the diagnosis HZ were on sick leave for an average length of 51 days, based on information from the SSIA. In addition, approximately 8,500 patients were on sick leave less than 14 days with an average length of 3.4 days. The major societal economic consequence of sick leave is cost for lost productivity as presented in Table 5. Out of the 22,800 HZ patients in working age with reported sick leave (20–74 years) 13,800 were employed during 2011.

**Table 5:** Number of sick leave days (<14 days and > =14 days) and productivity losses during 2011 for HZ

Age groups (years)	Number of employed HZ patients* (> = 50 years)	Number of HZ patients on sick leave**	Lost production cost (SEK)*** (> = 50 years)
Sick leave > =14 days	13 822 (6 689)	370	32 876 300 (15 909 500)
Sick leave <14 days	13 822 (6 689)	8 476	30 321 300 (24 119 200)
Total:			63 197 600 (40 028 700)

*Statistics Sweden; **Swedish Social Insurance Agency; ***Patients were off sick on average 51 days if sick leave > =14 days and 3.38 days if <14 days

The total sick leave cost for all patients during 2011 was estimated to 63.2 MSEK (7.3 M€) (40.0 MSEK (4.6 M€) for patients > =50 years).

### Total costs

In Sweden, during 2011, the overall societal cost of HZ and PHN was estimated to 227 MSEK (26.2 M€) including patients at all ages which corresponds to on average 7,600 SEK (€880) per HZ patient. If only patients 50 years and older were included the overall cost was estimated to 74 % of the costs (168 MSEK; 19.5 M€). The main contributors to the total cost for the treatment of HZ patients were primary care (43 %); sick leave (28 %); hospitalization (10 %) and specialist care (7 %). Medication was a relatively small contributor with 4 % (8.5 MSEK; 0.99 M€) to the overall costs for patients at all ages.

### Sensitivity analysis

To test how sensitive the overall cost was to changes in the estimated number of short term sick leave days these were increased with 50 % from 3.4 days to 5.1 days per employed HZ patient and year. The total cost increased from 227 MSEK (26.2 M€) in the base case to 242 MSEK (6 % increase; 28.3 M€). Sensitivity analyses were also conducted for a few other estimated parameters: increasing the GP consultation cost with 25 % increased the total cost with 8 % to 245 MSEK (28.6 M€); increasing the daily hospitalization cost with 25 % increased the total cost with 4 % to 235 MSEK (27.2 M€) and similar for the drug cost increased the total cost slightly to 229 MSEK (26.5 M€).

## Discussion

During 2011, almost 30,000 patients were diagnosed with HZ/PHN and the societal costs to treat these patients, including the costs to treat patients who later developed PHN, added up to nearly 227 MSEK (26.2 M€). The corresponding cost when only patients 50 years and older were considered adds to 168 MSEK (19.5 M€). In the study by Studahl et al. [8] it was estimated that just above 28,000 patients received antiviral drugs during 2010. They also demonstrated evidence of an increasing number of prescriptions of antiviral drugs (and diagnoses of HZ) from 2006 to 2010 in Sweden.

A number of studies have studied the cost of HZ and PHN covering different components and populations with a cost per patient range from 1,000 SEK (€116) to over 20,000 SEK (€2,300). In Spain, during 2006–2007 a study published by Cebrían-Cuenca et al. [28] estimated the cost per HZ patient from a societal perspective to €378 (around 3,300 SEK). In this study, the most important contributing cost factors were medications, followed by primary care physician visits, accounting for 37 % and 23 % of the societal costs, respectively. No hospitalizations were considered in this study. Gauthier et al. [14] estimated the combined mean total cost of outpatient and inpatient health care to £103 (1,042 SEK or €122) per HZ case. In addition, the cost per PHN episode was estimated to £397 (3,997 SEK or €466). Most of the costs were attributed to primary care (74 %). In an Italian retrospective, population-based study [29] the costs per HZ and PHN episodes were estimated. The costs associated with inpatient and outpatient care per HZ case were €2,592 per patient (22,419 SEK) compared to €123 per patient (1,064 SEK), respectively. The corresponding figures per PHN case were €2,806 (24,270 SEK) and €446 (3,858 SEK), respectively. Scott et al. [30] showed that the medical cost were highest in those aged over 65 years and societal costs highest in those aged under 65 years. However, the overall cost of HZ during the first six months was estimated at £524 per patient (in 2003 UK£; 4,489 SEK or €524). In that study, it was also suggested from a regression analysis that increasing age, the presence of immune compromising conditions and the use of antivirals were associated with increased costs to health service while a previous history of HZ was associated with decreased medical costs. Finally, in a French study by Mick et al. [31] the cost per patient for the treatment of patients, older than 50 years, with HZ and PHN was estimated to €932 (8,061 SEK) which corresponds to what was found in our study (8,240 SEK per patient).

As different approaches to estimate the cost of HZ and PHN have been used in all of the above studies, direct comparisons with our results are not straightforward. A couple of the studies (Gauthier et al. [14] and Gialloreti et al. [29]) indicated that primary care was the main cost contributor which we also found in our study. Cebrían-Cuenca et al. [28] estimated the cost per patient to 3,200 SEK (€370) per year (without hospitalizations) while Scott et al. [30] reported 4,489 SEK (€519) per six months. Both studies are in line with the 7,600 SEK (€879) per patient per year in our study.

The current study includes both direct and indirect cost which was not the case in all of the studies above. It is also a strength of the current study that register data, covering most of Sweden, were possible to collect for inpatient care, outpatient specialist care, prescribed medications and long term sick leave for HZ and PHN patients. A limitation of the current study, is the number of patient in inpatient care that has been estimated from ICD-10 code for HZ as the primary cause. When including patients also with HZ as the secondary cause, the number of hospitalizations increase by approximately 90 % [8]. Another limitation is that regional data (i.e., the number of diagnostic tests from Stockholm county council and outpatient primary care from Västra Götaland county council) has been extrapolated to the whole of Sweden. However, there is no data indicating large regional differences in HZ. Therefore, it is likely that the primary care cost estimated for Sweden is reliable.

In line with the findings by Jumaan et al. [12] and Pérez-Farinós et al. [13] our study showed that both the incidence and burden of HZ was larger for patients above 50 years old.

There is a risk that the incidence has been over-estimated since it was derived from the prescriptions of antiviral drugs in specified package sizes, which also can be prescribed to patients with other diagnoses than HZ. However, as postulated by Studahl et al. [8] it is likely that the number of diagnosed HZ patients exceeds the number prescriptions.

A very low mortality was identified due to HZ with only 11 fatalities identified. Considering the minimum yearly incidence of VZV central nervous system (CNS) disease of 1.8 per 100,000 with a significant mortality in encephalitic cases and reports of cerebral vasculitis complications due to VZV in the mistaken for common stroke, this might be an underestimate of the true VZV associated both morbidity and mortality [32, 33].

To the best of our knowledge no registry in Sweden contains data for short term sick leave i.e., shorter than 14 days. The assumption made here was 3.4 days per worker patient based on Drolet et al. [23]. Sensitivity analysis showed that the total cost increased with 6 % when the number of short term sick leave days was increased with 50 %. As the actual number of short term sick-leave days is not available in Sweden, future studies could benefit from acquiring more precise data for this variable. Moreover, in the current study it was decided not to include the cost for patients to travel to the physician or the hospital in our analysis. This would have included the cost for public transportation or the cost for travel by car to the nearest hospital or primary care center. Since the incidence of HZ/PHN increases with age many patients are old and may need help by an accompanying person when they visit the physician. This person is often a relative who needs to take time off from work to accompany the patient. The societal cost for productivity losses for the carer has not been taken into account in this study. Although the travel and carer cost most likely would not be insignificant, the estimations would have been based on crude assumptions, e.g., distance to hospital, type of transportation and who the average carer is. Since no real data was available to support any assumptions it was decided to exclude the transportation costs and the productivity loss of the carer in this study.

It was found that a considerable part of the societal cost for the treatment of HZ and PHN (168 MSEK (19.2 M€) corresponds to 74 %) are incurred by patients 50 years and older (corresponding to 78 % of the cases of HZ and PHN). Zostavax, a high-titer lived attenuated virus vaccine, is indicated for the prevention of HZ and PHN in adults 50 years of age and older in the EU. Its efficacy has been tested in a large randomized, double-blind, placebo-controlled trials (Shingles Prevention Study, SPS) [34]. This study reported that the vaccination reduced the incidence of HZ by 51.1 % and of PHN by 66.5 % in 38,546 immune competent adults aged 60 and older over a mean surveillance period of 3.1 years. It reduced the burden of illness due to HZ by 61.1 %, in that vaccinated individuals who developed HZ had a shorter duration, reduced disease severity and discomfort from pain compared to individuals from the placebo group who developed HZ [34]. The effect of the vaccine was confirmed by Schmader et al. [35] in the Zostavax Efficacy and Safety Trial (ZEST), where the incidence of HZ was reduced significantly (vaccine efficacy of 69.8 %) in patients between 50–59 years old and was well tolerated.

## Conclusions

The clinical and economic burden of HZ and PHN is significant in Sweden and the current study estimates that 74 % of the total costs are incurred by patients 50 years and older. Therefore, there is a potential for reducing the societal burden of these diseases through vaccination programs targeted at this group of patients that is likely to increase in the future. Although there are costs associated with a vaccination program, these need to be balanced against the likely reduction in disease burden and potential improvement of quality-of-life for many patients.

## Abbreviations

VZV: Varicella-zoster virus
HZ: Herpes zoster
PHN: Post-herpetic neuralgia
ICD-10: International classification of diseases
KPP: Cost per patient database
GP: General practitioner
VGR: Västra Götaland county council
SSIA: Swedish Social Insurance Agency
